# Supplementary material for: The voltage-gated Ca2+ channel subunit α2δ-4 regulates locomotor behavior and sensorimotor gating in mice
Source: PLoS One. 2022 Mar 30;17(3):e0263197. doi: 10.1371/journal.pone.0263197 (PMC8967030; doi:10.1371/journal.pone.0263197)
Supplement: S1 File — (PDF) [file pone.0263197.s001.pdf]

**Table 1**										
Mouse Weight										
Age (Days)	Genotype	Sex	Weight (g)				WT F	KO F	WT M	KO M
121	WT	F	22			Mean	21.63636	22.51818	29.55	29.28182
121	WT	F	23.4		Weight	SEM	6.523609	6.789487	9.34453	8.8288
126	WT	F	20.7							
126	WT	F	19.9							
108	WT	F	23.4							
108	WT	F	21.3							
108	WT	F	22.8							
121	WT	F	24							
121	WT	F	22							
104	WT	F	18.5							
104	WT	F	20							
110	KO	F	24.4							
110	KO	F	22.1							
110	KO	F	21.5							
110	KO	F	27.8							
110	KO	F	26.6							
121	KO	F	22							
121	KO	F	20.7							
121	KO	F	20.4							
121	KO	F	20.7							
131	KO	F	21.7							
131	KO	F	19.8							
156	WT	M	26.1							
156	WT	M	25.1							
156	WT	M	27.5							
170	WT	M	35.1							
170	WT	M	30.4							
170	WT	M	32.9							
170	WT	M	33.1							
156	WT	M	28.4							
156	WT	M	29.2							
156	WT	M	27.7							
145	KO	M	31.1							
145	KO	M	28.7							
145	KO	M	29.4							
143	KO	M	33							
143	KO	M	29.9							
143	KO	M	27.6							
143	KO	M	31.6							
143	KO	M	27							
143	KO	M	30.9							
143	KO	M	25.8							
143	KO	M	27.1							

FIGURE 1														
Mouse	Genotype	Sex	Trial Type	PPI	Block	Startle					WT F	KO F	WT M	KO M
601	WT	F	4dB	21	1	154.8333				Mean	-10.3636	-12.1818	-11	-1.54545
602	WT	F	4dB	-46	1	185			4dB PPI	SEM	-3.12475	-3.67296	-3.47851	-0.46597
603	WT	F	4dB	-74	1	147.6667				Mean	25.18182	6.363636	45	21.72727
604	WT	F	4dB	-7	1	411.1667			8dB PPI	SEM	7.592604	1.918709	14.23025	6.551019
612	WT	F	4dB	-26	1	74				Mean	57.18182	38.81818	73.4	46.90909
613	WT	F	4dB	43	1	155.6667		1B	16dB PPI	SEM	17.24097	11.70412	23.21112	14.14362
614	WT	F	4dB	-9	1	146.8333				Mean	229.9697	711.9698	583.5833	1642.864
615	WT	F	4dB	-20	1	583.6667			4dB Startle	SEM	69.33847	214.667	184.5452	495.342
616	WT	F	4dB	-20	1	136.5				Mean	195.9309	531.8955	356.5917	1382.318
617	WT	F	4dB	5	1	371.8333			8dB Startle	SEM	59.07539	160.3725	112.7642	416.7846
618	WT	F	4dB	19	1	162.5			16dB	Mean	272.303	642.3031	318.8	1211.106
605	KO	F	4dB	2	1	617.5		1C	Startle	SEM	82.10246	193.6617	100.8134	365.1622
606	KO	F	4dB	21	1	652.8333								
607	KO	F	4dB	-42	1	671.8333								
608	KO	F	4dB	-28	1	834.6667								
609	KO	F	4dB	-12	1	443								
610	KO	F	4dB	-3	1	556.5								
611	KO	F	4dB	-15	1	436.1667								
619	KO	F	4dB	-23	1	337.3333								
620	KO	F	4dB	-13	1	1250.667								
621	KO	F	4dB	-9	1	516.5								
622	KO	F	4dB	-12	1	1514.667								
628	WT	M	4dB	-2	1	387.6667								
629	WT	M	4dB	-9	1	263.6667								
630	WT	M	4dB	-120	1	322.8333								
631	WT	M	4dB	36	1	290.5								
632	WT	M	4dB	36	1	515.3333								
636	WT	M	4dB	22	1	354.3333								
637	WT	M	4dB	-11	1	1162.333								
638	WT	M	4dB	-24	1	364								
639	WT	M	4dB	-44	1	1281								
640	WT	M	4dB	6	1	894.1667								
623	KO	M	4dB	-2	1	1785.833								
624	KO	M	4dB	-2	1	1064.667								
625	KO	M	4dB	1	1	1021								
626	KO	M	4dB	-9	1	1087.667								
627	KO	M	4dB	4	1	1671.5								
633	KO	M	4dB	-16	1	1326.333								
634	KO	M	4dB	2	1	2491								
635	KO	M	4dB	1	1	2486.833								
641	KO	M	4dB	-13	1	1160								
642	KO	M	4dB	1	1	1930.833								
643	KO	M	4dB	16	1	2045.833								
601	WT	F	8dB	16	2	160.58								
602	WT	F	8dB	2	2	218.42								

603	WT	F	8dB	21	2	132								
604	WT	F	8dB	18	2	231.33								
612	WT	F	8dB	12	2	75								
613	WT	F	8dB	60	2	104.25								
614	WT	F	8dB	23	2	330.75								
615	WT	F	8dB	48	2	263.75								
616	WT	F	8dB	12	2	117.83								
617	WT	F	8dB	39	2	248								
618	WT	F	8dB	26	2	273.33								
605	KO	F	8dB	14	2	538.67								
606	KO	F	8dB	9	2	491.67								
607	KO	F	8dB	-15	2	468.33								
608	KO	F	8dB	-1	2	559.42								
609	KO	F	8dB	-11	2	441.67								
610	KO	F	8dB	27	2	503.75								
611	KO	F	8dB	10	2	318.92								
619	KO	F	8dB	6	2	419.25								
620	KO	F	8dB	2	2	474.92								
621	KO	F	8dB	18	2	542.5								
622	KO	F	8dB	11	2	1091.75								
628	WT	M	8dB	52	2	317.9167								
629	WT	M	8dB	36	2	248.3333								
630	WT	M	8dB	43	2	174.1667								
631	WT	M	8dB	56	2	249.25								
632	WT	M	8dB	73	2	301.75								
636	WT	M	8dB	41	2	252.1667								
637	WT	M	8dB	31	2	647.25								
638	WT	M	8dB	41	2	177.5								
639	WT	M	8dB	30	2	469.8333								
640	WT	M	8dB	47	2	727.75								
623	KO	M	8dB	15	2	1266.833								
624	KO	M	8dB	19	2	744.8333								
625	KO	M	8dB	24	2	969.8333								
626	KO	M	8dB	19	2	1079.417								
627	KO	M	8dB	11	2	1571								
633	KO	M	8dB	37	2	803.5833								
634	KO	M	8dB	39	2	1659								
635	KO	M	8dB	24	2	1985.25								
641	KO	M	8dB	20	2	770.25								
642	KO	M	8dB	16	2	1849.667								
643	KO	M	8dB	15	2	2505.833								
601	WT	F	16dB	65	3	195.8333								
602	WT	F	16dB	48	3	398.1667								
603	WT	F	16dB	68	3	210.6667								
604	WT	F	16dB	78	3	259.1667								
612	WT	F	16dB	50	3	73.33333								
613	WT	F	16dB	59	3	75.33333								

614	WT	F	16dB	49	3	381.8333								
615	WT	F	16dB	53	3	493								
616	WT	F	16dB	44	3	76.16667								
617	WT	F	16dB	73	3	216.6667								
618	WT	F	16dB	42	3	615.1667								
605	KO	F	16dB	11	3	402.6667								
606	KO	F	16dB	43	3	228.6667								
607	KO	F	16dB	37	3	607.8333								
608	KO	F	16dB	28	3	791								
609	KO	F	16dB	27	3	513.5								
610	KO	F	16dB	39	3	643								
611	KO	F	16dB	43	3	560.3333								
619	KO	F	16dB	69	3	504.1667								
620	KO	F	16dB	29	3	963.6667								
621	KO	F	16dB	53	3	490.3333								
622	KO	F	16dB	48	3	1360.167								
628	WT	M	16dB	81	3	332.5								
629	WT	M	16dB	73	3	200.6667								
630	WT	M	16dB	63	3	119.3333								
631	WT	M	16dB	69	3	115.6667								
632	WT	M	16dB	80	3	181								
636	WT	M	16dB	72	3	252.5								
637	WT	M	16dB	73	3	892.5								
638	WT	M	16dB	67	3	98.16667								
639	WT	M	16dB	77	3	580.8333								
640	WT	M	16dB	79	3	414.8333								
623	KO	M	16dB	31	3	991.6667								
624	KO	M	16dB	35	3	742.8333								
625	KO	M	16dB	45	3	849								
626	KO	M	16dB	25	3	1380.667								
627	KO	M	16dB	38	3	1760								
633	KO	M	16dB	67	3	650								
634	KO	M	16dB	59	3	1544								
635	KO	M	16dB	64	3	1869.833								
641	KO	M	16dB	63	3	1045.333								
642	KO	M	16dB	49	3	1148								
643	KO	M	16dB	40	3	1340.833								

FIGURE 2												
Mouse	Genotype	Sex	Trial Type	Threshold					WT M	KO M	WT F	KO F
A1	WT	M	8kHz	20				Mean	20.9375	26.5625	30	24.6875
A2	WT	M	8kHz	20			8kHz	SEM	6.312894	8.008895	9.04534	7.443561
A3	WT	M	8kHz	20				Mean	20.3125	23.125	28.125	21.25
A4	WT	M	8kHz	25			16kHz	SEM	6.124449	6.97245	8.480007	6.407116
A5	WT	M	8kHz	20				Mean	20	22.5	26.875	20
A6	WT	M	8kHz	20			24kHz	SEM	6.030227	6.784005	8.103117	6.030227
A7	WT	M	8kHz	20				Mean	20.3125	25	33.75	20
A8	WT	M	8kHz	20			32kHz	SEM	6.124449	7.537784	10.17601	6.030227
A9	WT	M	8kHz	20				Mean	27.8125	37.8125	41.25	38.125
A10	WT	M	8kHz	25		2	Click	SEM	8.385784	11.4009	12.43734	11.49512
A11	WT	M	8kHz	20								
A12	WT	M	8kHz	25								
A13	WT	M	8kHz	20								
A14	WT	M	8kHz	20								
A15	WT	M	8kHz	20								
A16	WT	M	8kHz	20								
A17	KO	M	8kHz	25								
A18	KO	M	8kHz	25								
A19	KO	M	8kHz	30								
A20	KO	M	8kHz	30								
A21	KO	M	8kHz	45								
A22	KO	M	8kHz	50								
A23	KO	M	8kHz	30								
A24	KO	M	8kHz	25								
A25	KO	M	8kHz	20								
A26	KO	M	8kHz	20								
A27	KO	M	8kHz	20								
A28	KO	M	8kHz	20								
A29	KO	M	8kHz	25								
A30	KO	M	8kHz	20								
A31	KO	M	8kHz	20								
A32	KO	M	8kHz	20								
A1	WT	M	16kHz	20								
A2	WT	M	16kHz	20								
A3	WT	M	16kHz	20								
A4	WT	M	16kHz	20								
A5	WT	M	16kHz	25								
A6	WT	M	16kHz	20								
A7	WT	M	16kHz	20								
A8	WT	M	16kHz	20								
A9	WT	M	16kHz	20								
A10	WT	M	16kHz	20								
A11	WT	M	16kHz	20								
A12	WT	M	16kHz	20								
A13	WT	M	16kHz	20								

A14	WT	M	16kHz	20									
A15	WT	M	16kHz	20									
A16	WT	M	16kHz	20									
A17	KO	M	16kHz	20									
A18	KO	M	16kHz	20									
A19	KO	M	16kHz	25									
A20	KO	M	16kHz	20									
A21	KO	M	16kHz	40									
A22	KO	M	16kHz	45									
A23	KO	M	16kHz	20									
A24	KO	M	16kHz	20									
A25	KO	M	16kHz	20									
A26	KO	M	16kHz	20									
A27	KO	M	16kHz	20									
A28	KO	M	16kHz	20									
A29	KO	M	16kHz	20									
A30	KO	M	16kHz	20									
A31	KO	M	16kHz	20									
A32	KO	M	16kHz	20									
A1	WT	M	24kHz	20									
A2	WT	M	24kHz	20									
A3	WT	M	24kHz	20									
A4	WT	M	24kHz	20									
A5	WT	M	24kHz	20									
A6	WT	M	24kHz	20									
A7	WT	M	24kHz	20									
A8	WT	M	24kHz	20									
A9	WT	M	24kHz	20									
A10	WT	M	24kHz	20									
A11	WT	M	24kHz	20									
A12	WT	M	24kHz	20									
A13	WT	M	24kHz	20									
A14	WT	M	24kHz	20									
A15	WT	M	24kHz	20									
A16	WT	M	24kHz	20									
A17	KO	M	24kHz	20									
A18	KO	M	24kHz	20									
A19	KO	M	24kHz	20									
A20	KO	M	24kHz	20									
A21	KO	M	24kHz	40									
A22	KO	M	24kHz	40									
A23	KO	M	24kHz	20									
A24	KO	M	24kHz	20									
A25	KO	M	24kHz	20									
A26	KO	M	24kHz	20									
A27	KO	M	24kHz	20									
A28	KO	M	24kHz	20									

A29	KO	M	24kHz	20								
A30	KO	M	24kHz	20								
A31	KO	M	24kHz	20								
A32	KO	M	24kHz	20								
A1	WT	M	32kHz	20								
A2	WT	M	32kHz	20								
A3	WT	M	32kHz	20								
A4	WT	M	32kHz	20								
A5	WT	M	32kHz	20								
A6	WT	M	32kHz	20								
A7	WT	M	32kHz	25								
A8	WT	M	32kHz	20								
A9	WT	M	32kHz	20								
A10	WT	M	32kHz	20								
A11	WT	M	32kHz	20								
A12	WT	M	32kHz	20								
A13	WT	M	32kHz	20								
A14	WT	M	32kHz	20								
A15	WT	M	32kHz	20								
A16	WT	M	32kHz	20								
A17	KO	M	32kHz	20								
A18	KO	M	32kHz	20								
A19	KO	M	32kHz	20								
A20	KO	M	32kHz	20								
A21	KO	M	32kHz	35								
A22	KO	M	32kHz	40								
A23	KO	M	32kHz	20								
A24	KO	M	32kHz	20								
A25	KO	M	32kHz	40								
A26	KO	M	32kHz	35								
A27	KO	M	32kHz	25								
A28	KO	M	32kHz	20								
A29	KO	M	32kHz	20								
A30	KO	M	32kHz	25								
A31	KO	M	32kHz	20								
A32	KO	M	32kHz	20								
A33	WT	F	8kHz	20								
A34	WT	F	8kHz	25								
A35	WT	F	8kHz	20								
A36	WT	F	8kHz	20								
A37	WT	F	8kHz	20								
A38	WT	F	8kHz	20								
A39	WT	F	8kHz	20								
A40	WT	F	8kHz	20								
A41	WT	F	8kHz	20								
A42	WT	F	8kHz	25								
A43	WT	F	8kHz	65								

A44	WT	F	8kHz	70									
A45	WT	F	8kHz	30									
A46	WT	F	8kHz	25									
A47	WT	F	8kHz	40									
A48	WT	F	8kHz	40									
A49	KO	F	8kHz	45									
A50	KO	F	8kHz	45									
A51	KO	F	8kHz	20									
A52	KO	F	8kHz	20									
A53	KO	F	8kHz	20									
A54	KO	F	8kHz	20									
A55	KO	F	8kHz	25									
A56	KO	F	8kHz	25									
A57	KO	F	8kHz	20									
A58	KO	F	8kHz	25									
A59	KO	F	8kHz	20									
A60	KO	F	8kHz	20									
A61	KO	F	8kHz	25									
A62	KO	F	8kHz	25									
A63	KO	F	8kHz	20									
A64	KO	F	8kHz	20									
A33	WT	F	16kHz	20									
A34	WT	F	16kHz	20									
A35	WT	F	16kHz	20									
A36	WT	F	16kHz	20									
A37	WT	F	16kHz	20									
A38	WT	F	16kHz	20									
A39	WT	F	16kHz	20									
A40	WT	F	16kHz	20									
A41	WT	F	16kHz	20									
A42	WT	F	16kHz	20									
A43	WT	F	16kHz	65									
A44	WT	F	16kHz	70									
A45	WT	F	16kHz	20									
A46	WT	F	16kHz	25									
A47	WT	F	16kHz	40									
A48	WT	F	16kHz	30									
A49	KO	F	16kHz	30									
A50	KO	F	16kHz	30									
A51	KO	F	16kHz	20									
A52	KO	F	16kHz	20									
A53	KO	F	16kHz	20									
A54	KO	F	16kHz	20									
A55	KO	F	16kHz	20									
A56	KO	F	16kHz	20									
A57	KO	F	16kHz	20									
A58	KO	F	16kHz	20									

A59	KO	F	16kHz	20								
A60	KO	F	16kHz	20								
A61	KO	F	16kHz	20								
A62	KO	F	16kHz	20								
A63	KO	F	16kHz	20								
A64	KO	F	16kHz	20								
A33	WT	F	24kHz	20								
A34	WT	F	24kHz	20								
A35	WT	F	24kHz	20								
A36	WT	F	24kHz	20								
A37	WT	F	24kHz	20								
A38	WT	F	24kHz	20								
A39	WT	F	24kHz	20								
A40	WT	F	24kHz	20								
A41	WT	F	24kHz	20								
A42	WT	F	24kHz	25								
A43	WT	F	24kHz	60								
A44	WT	F	24kHz	60								
A45	WT	F	24kHz	25								
A46	WT	F	24kHz	20								
A47	WT	F	24kHz	40								
A48	WT	F	24kHz	20								
A49	KO	F	24kHz	20								
A50	KO	F	24kHz	20								
A51	KO	F	24kHz	20								
A52	KO	F	24kHz	20								
A53	KO	F	24kHz	20								
A54	KO	F	24kHz	20								
A55	KO	F	24kHz	20								
A56	KO	F	24kHz	20								
A57	KO	F	24kHz	20								
A58	KO	F	24kHz	20								
A59	KO	F	24kHz	20								
A60	KO	F	24kHz	20								
A61	KO	F	24kHz	20								
A62	KO	F	24kHz	20								
A63	KO	F	24kHz	20								
A64	KO	F	24kHz	20								
A33	WT	F	32kHz	25								
A34	WT	F	32kHz	25								
A35	WT	F	32kHz	30								
A36	WT	F	32kHz	25								
A37	WT	F	32kHz	20								
A38	WT	F	32kHz	20								
A39	WT	F	32kHz	20								
A40	WT	F	32kHz	20								
A41	WT	F	32kHz	20								

A42	WT	F	32kHz	25									
A43	WT	F	32kHz	65									
A44	WT	F	32kHz	65									
A45	WT	F	32kHz	40									
A46	WT	F	32kHz	40									
A47	WT	F	32kHz	60									
A48	WT	F	32kHz	40									
A49	KO	F	32kHz	20									
A50	KO	F	32kHz	20									
A51	KO	F	32kHz	20									
A52	KO	F	32kHz	20									
A53	KO	F	32kHz	20									
A54	KO	F	32kHz	20									
A55	KO	F	32kHz	20									
A56	KO	F	32kHz	20									
A57	KO	F	32kHz	20									
A58	KO	F	32kHz	20									
A59	KO	F	32kHz	20									
A60	KO	F	32kHz	20									
A61	KO	F	32kHz	20									
A62	KO	F	32kHz	20									
A63	KO	F	32kHz	20									
A64	KO	F	32kHz	20									
A1	WT	M	Click	30									
A2	WT	M	Click	30									
A3	WT	M	Click	25									
A4	WT	M	Click	25									
A5	WT	M	Click	20									
A6	WT	M	Click	20									
A7	WT	M	Click	10									
A8	WT	M	Click	20									
A9	WT	M	Click	35									
A10	WT	M	Click	35									
A11	WT	M	Click	30									
A12	WT	M	Click	30									
A13	WT	M	Click	30									
A14	WT	M	Click	30									
A15	WT	M	Click	40									
A16	WT	M	Click	35									
A17	KO	M	Click	40									
A18	KO	M	Click	35									
A19	KO	M	Click	35									
A20	KO	M	Click	40									
A21	KO	M	Click	60									
A22	KO	M	Click	65									
A23	KO	M	Click	35									
A24	KO	M	Click	30									

A25	KO	M	Click	35								
A26	KO	M	Click	30								
A27	KO	M	Click	30								
A28	KO	M	Click	35								
A29	KO	M	Click	35								
A30	KO	M	Click	30								
A31	KO	M	Click	35								
A32	KO	M	Click	35								
A33	WT	F	Click	35								
A34	WT	F	Click	35								
A35	WT	F	Click	35								
A36	WT	F	Click	40								
A37	WT	F	Click	30								
A38	WT	F	Click	30								
A39	WT	F	Click	30								
A40	WT	F	Click	35								
A41	WT	F	Click	25								
A42	WT	F	Click	30								
A43	WT	F	Click	90								
A44	WT	F	Click	90								
A45	WT	F	Click	30								
A46	WT	F	Click	40								
A47	WT	F	Click	45								
A48	WT	F	Click	40								
A49	KO	F	Click	50								
A50	KO	F	Click	50								
A51	KO	F	Click	35								
A52	KO	F	Click	30								
A53	KO	F	Click	30								
A54	KO	F	Click	30								
A55	KO	F	Click	40								
A56	KO	F	Click	40								
A57	KO	F	Click	45								
A58	KO	F	Click	40								
A59	KO	F	Click	45								
A60	KO	F	Click	50								
A61	KO	F	Click	25								
A62	KO	F	Click	20								
A63	KO	F	Click	35								
A64	KO	F	Click	45								

FIGURE 3																
Mouse	Genotype	Sex	EPM_O	EPM_C	LD_L	LD_D	OFT_I	OFT_O					WT F	KO F	WT M	KO M
601	WT	F			30	70	9.5	90.5				Mean	6.125	14.5	6.444444	13.27273
602	WT	F	4	78	24	76	5	95		3C	EPM Open	SEM	1.846757	4.371914	2.037912	4.001878
603	WT	F	18	55	32	68	17	83				Mean	67.75	56.3	72.44444	54.72727
604	WT	F			41	59	21	79		3D	EPM Closed	SEM	20.42739	16.97509	22.90894	16.50089
612	WT	F	7	69	21	79	25.5	74.5				Mean	29.81818	33.36364	37.8	51.09091
613	WT	F	2	74	30	70	8	92		3E	LD Light	SEM	8.99052	10.05951	11.95341	15.40449
614	WT	F	6	72	19	81	15.5	84.5				Mean	70.18182	33.36364	37.8	48.90909
615	WT	F	6	47	29	71	15.5	84.5		3F	LD Dark	SEM	21.16061	10.05951	11.95341	14.74665
616	WT	F	6	70	20	80	9.5	90.5				Mean	14.72727	11.27273	12.3	15.31818
617	WT	F	0	77	41	59	23.5	76.5		3A	OFT Inner	SEM	4.44044	3.398855	3.889602	4.618606
618	WT	F			41	59	12	88				Mean	85.27273	88.72727	87.7	84.68182
605	KO	F	16	70	22	78	11.5	88.5		3B	OFT Outer	SEM	25.71069	26.75228	27.73318	25.53253
606	KO	F			40	60	20.5	79.5								
607	KO	F	16	45	16	84	13	87								
608	KO	F	39	37	34	66	7.5	92.5								
609	KO	F	14	64	29	71	5	95								
610	KO	F	27	50	38	62	18	82								
611	KO	F	5	67	31	69	6	94								
619	KO	F	7	63	49	51	18.5	81.5								
620	KO	F	7	57	42	58	4	96								
621	KO	F	8	56	28	72	8	92								
622	KO	F	6	54	38	63	12	88								
628	WT	M	0	100	18	82	6	94								
629	WT	M	15	59	40	60	12	88								
630	WT	M	8	74	39	61	14	86								
631	WT	M	6	62	56	44	6	94								
632	WT	M	6	60	30	70	15	85								
636	WT	M	5	79	29	71	7.5	92.5								
637	WT	M	4	79	45	55	28	72								
638	WT	M	4	70	18	82	10	90								
639	WT	M	10	69	17	83	4	96								
640	WT	M			86	14	20.5	79.5								
623	KO	M	16	61	45	55	10	90								
624	KO	M	6	58	38	62	16.5	83.5								
625	KO	M	6	71	52	48	11	89								
626	KO	M	6	54	40	60	10	90								
627	KO	M	15	46	47	53	24	76								
633	KO	M	3	71	42	58	8.5	91.5								
634	KO	M	33	31	61	39	11.5	88.5								
635	KO	M	1	63	55	45	21.5	78.5								
641	KO	M	22	44	66	34	23	77								
642	KO	M	24	38	56	44	13	87								
643	KO	M	14	65	60	40	19.5	80.5								

Stress	FIGURE 4															
Mouse	Genotype	Sex	FST 1	FST 2	FST 3	FST 4	FST 5	FST 6					WT F	KO F	WT M	KO M
601	WT	F	5.85	9.4	13.1	10.45	2.2	12.7				Mean	5.014545	12.15909	1.763334	8.318185
602	WT	F	2.1	24.15	32	30.8	36.45	42.05			FST 1	SEM	1.511942	3.666104	0.557615	2.508027
603	WT	F	0.35	0.55	4.65	3.95	9.55	16.25				Mean	21.07545	30.38273	25.00001	21.86061
604	WT	F	1.95	17.1	8.35	6.75	11.25	16.55			FST 2	SEM	6.354489	9.160737	7.905698	6.591222
612	WT	F	6.57	28.03	34.3	38.27	40.4	43.4				Mean	25.57273	31.56727	33.67999	27.91818
613	WT	F	4.63	39.33	30.7	35.6	32.93	38.77			FST 3	SEM	7.710467	9.517891	10.65055	8.417647
614	WT	F	4.6	30.1	33.5	28.6	40.2	32.95				Mean	24.68909	31.56727	33.67999	30.16666
615	WT	F	6.9	20.6	36.57	49.43	49.17	46.53			FST 4	SEM	7.444041	9.517891	10.65055	9.095591
616	WT	F	4.57	15.43	19.53	17.1	31.4	28.93				Mean	28.11364	25.35182	31.34667	36.02726
617	WT	F	13.17	27.07	31.47	28.63	20.63	26.77			FST 5	SEM	8.47658	7.643861	9.912687	10.86263
618	WT	F	4.47	20.07	37.13	22	35.07	35.9				Mean	30.98182	29.54909	38.61717	31.53029
605	KO	F	19.05	34.5	40.4	39.85	32.4	48.05		4D-F	FST 6	SEM	9.34137	8.909386	12.21182	9.506741
606	KO	F	17.9	32.6	21	33.6	26.15	35.85								
607	KO	F	6	5	21	12.45	30.35	31.35								
608	KO	F	9.77	40.6	51.9	48.15	37.7	43.25								
609	KO	F	12.3	52.07	45.3	6.9	39.57	30.13								
610	KO	F	23.37	55.17	50.1	53.67	42.53	50.33								
611	KO	F	11.83	29.3	38.7	36.23	24.63	19.37								
619	KO	F	11.1	18.47	31.57	44.27	29.87	33.67								
620	KO	F	19.27	28.8	25.1	18.7	13.83	25.07								
621	KO	F	0.53	18.03	7.47	7.27	0.47	4.57								
622	KO	F	2.63	19.67	14.7	0.47	1.37	3.4								
628	WT	M	0.46667	7.53333	34.3333	38.4333	24.9667	15.3667								
629	WT	M	0.63333	0.1	3.1	11.5333	12.9333	27.9051								
630	WT	M	2.9	28.8	29.1	26.9667	24.6667	27.7667								
631	WT	M	0.96667	35.1667	43.2667	49.4333	42.4	45.3								
632	WT	M	0.56667	12.7667	34.5333	35.7333	27.8	37.3333								
636	WT	M	5.16667	31.1667	38.5	45.9	34.0333	48.3								
637	WT	M	0.83333	41.5667	40.5	46.7667	39.8333	49.5333								
638	WT	M	2.4	38	24.7333	48.3333	43.3667	42.0333								
639	WT	M	3.1	28.6667	50.9	49.5333	39.4667	51.9								
640	WT	M	0.6	26.2333	37.8333	34.4	24	40.7333								
623	KO	M	16.6	51.9667	50.5333	52	41.5333	46.1								
624	KO	M	17.2	38.7	40.3667	41.8667	31.6333	42.0333								
625	KO	M	2.16667	13.6	27.8	28.9333	36.3	42.9333								
626	KO	M	17.6	40.1	53.0333	48.3333	54.1667	50								
627	KO	M	13.8	8.26667	3.16667	25.6667	45.2333	17.9								
633	KO	M	2.86667	14.4	36.2	26.0333	31.3333	40.0333								
634	KO	M	0.9	8	21.8333	38.3	36.5	33.8333								
635	KO	M	0.56667	9.8	0.56667	0	26.4	25.5667								
641	KO	M	4.13333	26.7	27.8667	28.4667	34.8667	6.23333								
642	KO	M	12.7667	21.4	28.1	21.1333	30.4	20.4								
643	KO	M	2.9	7.53333	17.6333	21.1	27.9333	21.8								

	TST 1	TST 2	TST 3	TST 4	TST 5	TST 6					WT F	KO F	WT M	KO M
	6.8	43.7	31.8	30.7	17.65	42.4				Mean	9.801818	3.643636	1.763334	8.318185
	33	52.65	19	45.7	43.6	44.35			TST 1	SEM	2.955359	1.098598	0.557615	2.508027
	14.35	32.65	44.2	44	35.4	53.75				Mean	27.91545	17.79455	25.00001	21.86061
	1.7	25.2	25.3	44.5	49.45	46.25			TST 2	SEM	8.416826	5.365257	7.905698	6.591222
	0.7	27.1	48.03	47.4	40.77	47.3				Mean	28.88455	24.94545	33.67999	27.91818
	4.77	28.27	33.67	47.03	44.43	46.73			TST 3	SEM	8.709018	7.521338	10.65055	8.417647
	2.43	14.23	29.5	45.57	51.47	30.7				Mean	39.27909	24.94545	33.67999	30.16666
	5.37	31.87	29	41.93	41.87	38.4			TST 4	SEM	11.84309	7.521338	10.65055	9.095591
	14.23	42.9	41.53	31	46.23	41.47				Mean	41.50273	25.50455	31.34667	36.02726
	23.07	6.5	9.77	32.67	37.33	47.77			TST 5	SEM	12.51354	7.68991	9.912687	10.86263
	1.4	2	5.93	21.57	48.33	35.9				Mean	43.18364	28.38727	38.61717	31.53029
	3.4	14.2	22.5	26.4	25.3	23.9		4A-C	TST 6	SEM	13.02036	8.559085	12.21182	9.506741
	4.45	20.1	19	20	42.6	28.35								
	1.65	21.4	32.3	29.2	26.8	30.35								
	3.65	19.3	37.45	28	28.35	39.05								
	13.5	24.27	31.13	35.07	41.8	42.4								
	2.23	20.9	21.35	14.4	32.4	31.5								
	1	9.47	20.3	26.9	10.9	20.47								
	2.3	13.87	15.47	28	15.9	27.97								
	1.03	3.93	26.57	29.3	21.7	14.17								
	3.2	32.97	27.93	35.27	2.37	30.1								
	3.67	15.33	20.4	39.17	32.43	24								
	0.46667	7.53333	34.3333	38.4333	24.9667	15.3667								
	0.63333	0.1	3.1	11.5333	12.9333	27.9051								
	2.9	28.8	29.1	26.9667	24.6667	27.7667								
	0.96667	35.1667	43.2667	49.4333	42.4	45.3								
	0.56667	12.7667	34.5333	35.7333	27.8	37.3333								
	5.16667	31.1667	38.5	45.9	34.0333	48.3								
	0.83333	41.5667	40.5	46.7667	39.8333	49.5333								
	2.4	38	24.7333	48.3333	43.3667	42.0333								
	3.1	28.6667	50.9	49.5333	39.4667	51.9								
	0.6	26.2333	37.8333	34.4	24	40.7333								
	16.6	51.9667	50.5333	52	41.5333	46.1								
	17.2	38.7	40.3667	41.8667	31.6333	42.0333								
	2.16667	13.6	27.8	28.9333	36.3	42.9333								
	17.6	40.1	53.0333	48.3333	54.1667	50								
	13.8	8.26667	3.16667	25.6667	45.2333	17.9								
	2.86667	14.4	36.2	26.0333	31.3333	40.0333								
	0.9	8	21.8333	38.3	36.5	33.8333								
	0.56667	9.8	0.56667	0	26.4	25.5667								
	4.13333	26.7	27.8667	28.4667	34.8667	6.23333								
	12.7667	21.4	28.1	21.1333	30.4	20.4								
	2.9	7.53333	17.6333	21.1	27.9333	21.8								

FIGURE 5													
Mouse	Genotype	Sex	OFT_DT	EPM_DT	LD_DT	Rotarod_L					WT F	KO F	WT M
601	WT	F	15.725		3.9182	410.64			OFT	Mean	17.77555	25.35455	19.0652
602	WT	F	15.902	8.986	3.4317	389.76		5B	Distance	SEM	5.359529	7.644683	6.028946
603	WT	F	16.478	6.85	3.1057	257.52			EPM	Mean	8.33475	12.2937	9.855222
604	WT	F	20.936		4.6667	522		5C	Distance	SEM	2.513022	3.70669	3.116495
612	WT	F	13.926	9.392	2.4756	361.92			LD	Mean	3.647591	5.423109	3.556191
613	WT	F	16.046	8.205	3.4185	445.44		5D	Distance	SEM	1.09979	1.635129	1.124566
614	WT	F	15.237	5.882	3.9636	348			Rotarod	Mean	365.0836	5.423109	3.556191
615	WT	F	19.748	9.975	3.7697	292.32		5A	Latency	SEM	110.0769	1.635129	1.124566
616	WT	F	24.452	8.497	4.0281	334.08							
617	WT	F	13.889	8.891	3.9945	313.2							
618	WT	F	23.192		3.3512	341.04							
605	KO	F	33.128	8.432	4.6786	222.72							
606	KO	F	26.907		5.503	250.56							
607	KO	F	19.432	11.56	9.2208	222.72							
608	KO	F	23.851	16.089	5.3607	375.84							
609	KO	F	22.343	13.751	5.2151	354.96							
610	KO	F	15.717	12.109	4.4586	257.52							
611	KO	F	17.064	13.398	5.2311	452.4							
619	KO	F	41.268	10.883	3.1137	334.08							
620	KO	F	24.551	10.975	7.3099	174							
621	KO	F	25.826	13.377	3.92	236.64							
622	KO	F	28.813	12.363	5.6427	187.92							
628	WT	M	19.768	7.18	2.993992	208.8							
629	WT	M	27.08	12.929	3.957801	236.64							
630	WT	M	21.624	10.072	4.285092	180.96							
631	WT	M	18.008	8.972	4.633156	187.92							
632	WT	M	22.707	9.917	4.042484	306.24							
636	WT	M	18.455	7.732	3.182917	174							
637	WT	M	13.413	7.774	4.088989	403.68							
638	WT	M	20.212	11.528	2.898943	201.84							
639	WT	M	15.086	12.593	3.066585	201.84							
640	WT	M	14.299		2.411955	139.2							
623	KO	M	16.161	12.679	3.5519	153.12							
624	KO	M	27.715	9.977	5.138837	264.48							
625	KO	M	16.92	10.468	4.033287	250.56							

626	KO	M	19.633	8.991	5.491843	146.16							
627	KO	M	18.602	7.245	5.103566	187.92							
633	KO	M	33.345	11.014	4.695774	264.48							
634	KO	M	22.919	12.611	6.551157	160.08							
635	KO	M	74.143	10.701	8.37129	257.52							
641	KO	M	53.207	15.125	11.73488	132.24							
642	KO	M	24.723	11.857	5.507325	118.32							
643	KO	M	35.692	12.481	6.44346	264.48							

KO M
31.18727
9.403317
11.19536
3.375529
6.056665
1.826153
199.9418
60.28473
